# Immunological Response to COVID-19 Vaccination in Ovarian Cancer Patients Receiving PARP Inhibitors

**DOI:** 10.3390/vaccines9101148

**Published:** 2021-10-08

**Authors:** Michalis Liontos, Evangelos Terpos, Christos Markellos, Flora Zagouri, Alexandros Briasoulis, Ioanna Katsiana, Efthymia Skafida, Oraianthi Fiste, Elena Kunadis, Angeliki Andrikopoulou, Maria Kaparelou, Konstantinos Koutsoukos, Maria Gavriatopoulou, Efstathios Kastritis, Ioannis P. Trougakos, Meletios-Athanasios Dimopoulos

**Affiliations:** 1 Department of Clinical Therapeutics, School of Medicine, Alexandra General Hospital, National and Kapodistrian University of Athens, 11528 Athens, Greece; mliontos@gmail.com (M.L.); eterpos@med.uoa.gr (E.T.); chrismarkellos@hotmail.com (C.M.); alexbriasoulis@gmail.com (A.B.); iwannakatsiana@gmail.com (I.K.); skafidaefi@gmail.com (E.S.); oraianthifiste@gmail.com (O.F.); kounadielena@gmail.com (E.K.); aggandrikop@med.uoa.gr (A.A.); mkaparelou@yahoo.com (M.K.); koutsoukos.k@gmail.com (K.K.); mgavria@med.uoa.gr (M.G.); ekastritis@med.uoa.gr (E.K.); mdimop@uoa.gr (M.-A.D.); 2 Department of Cell Biology and Biophysics, Faculty of Biology, National and Kapodistrian University of Athens, 11528 Athens, Greece; itrougakos@biol.uoa.gr

**Keywords:** ovarian cancer, SARS-CoV-2, vaccination, PARP inhibitors

## Abstract

Objective: Vaccination for SARS-CoV-2 provides significant protection against the infection in the general population. However, limited data exist for cancer patients under systemic therapy. Methods: In this cohort, we prospectively enrolled cancer patients treated with PARPi as well as healthy volunteers in order to study the kinetics of anti-SARS-CoV-2 antibodies (NAbs) after COVID-19 vaccination. Baseline demographics, co-morbidities, and NAb levels were compared between the two groups. Results: The results of the cohort of 36 patients receiving PARP inhibitors are presented here. Despite no new safety issues being noticed, their levels of SARS-CoV-2 neutralizing antibodies were significantly lower in comparison to matched healthy volunteers up to day 30 after the second dose. Conclusions: These results suggest that maintaining precautions against COVID-19 is essential for cancer patients and should be taken into consideration for the patients under treatment, while further exploration is needed to reduce the uncertainty of SARS-CoV-2 immunity among cancer patients under treatment.

## 1. Introduction

Cancer is a major public health issue worldwide. It is estimated that almost 20 million new cancer cases and 10.0 million cancer deaths occurred in 2020 [1]. The COVID-19 pandemic, caused by severe acute respiratory syndrome coronavirus 2 (SARS-CoV-2), has had an unprecedented impact throughout the world. COVID-19 patients mainly present with symptoms from the respiratory system, but other organs may be affected as well. Disease severity varies, from entirely asymptomatic carriers to patients with severe respiratory failure and multiple complications due to SARS-CoV-2 infection that lead to multiple organ failure and death [2]. Patients with cancer are more vulnerable to COVID-19, as shown by retrospectively analyzed cohorts of patients in several countries [3]. As a risk factor, cancer increased mortality among COVID-19 patients. Under this perspective, delays in cancer treatment (including surgical treatment, radiotherapy, and chemotherapy), in regular follow-ups of patients, as well as in screening procedures have been noted [4]. Therefore, cancer patients have been a key target group of the vaccination strategy in most countries and have been prioritized to receive COVID-19 vaccination in several countries, including Greece. However, patients with cancer were excluded from SARS-CoV-2 vaccine registrational trials and data regarding the safety and efficacy of vaccination in this population are lacking [5,6]. 

Poly (ADP-ribose) polymerase inhibitors (PARPi) inhibit enzymatic activity and trap PARP proteins on DNA, leading to cancer cell death [7]. PARPi have transformed the management of many common cancer types. These agents are currently used in ovarian cancer patients as a maintenance treatment after the completion of chemotherapy for newly diagnosed or recurrent disease. PARPi have also been suggested by regulatory authorities to be used to discover additional biomarker-driven indications in breast, prostate, and pancreatic cancer [8]. Preclinical studies have also indicated that these agents could have an immunomodulatory effect, modulating both the innate and adaptive immune system [9].

Under this perspective, we undertook a large prospective study (NCT04743388), enrolling patients with solid cancers and hematologic malignancies as well as healthy volunteers, in order to study the kinetics of anti-SARS-CoV-2 antibodies after COVID-19 vaccination [10]. Herein, we report the development of neutralizing antibodies (NAbs) against SARS-CoV-2 in patients with solid tumors receiving PARPi after vaccination with BNT162b2, AZD1222, or mRNA-1273 vaccines. 

## 2. Material and Methods

In the prospective study NCT04743388, we enrolled cancer patients and healthy volunteers in order to study the kinetics of anti-SARS-CoV-2 antibodies after COVID-19 vaccination. Major inclusion criteria for the cohort of patients treated with PARPi included: (i) age above 18 years; (ii) histologically confirmed ovarian/breast/prostate/pancreatic cancer under treatment with PARPi, irrespective of the treatment phase; and (iii) eligibility for vaccination. Blood from both patients and controls was collected on day 1 prior to vaccination, on day 22, and one month after the second vaccination dose. Serum was separated within 4 h of blood collection and stored at −80°C until the day of measurement. NAbs against SARS-CoV-2 were measured using an FDA-approved methodology (ELISA, cPass™ SARS-CoV-2 NAbs Detection Kit; GenScript, Piscataway, NJ, USA) [11] on the abovementioned timepoints. Samples of the same patient or control were measured in the same ELISA plate. The study was approved by the respective ethical committees (decision number 15/23-12-2020) in accordance with the Declaration of Helsinki and the International Conference on Harmonization for Good Clinical Practice. All patients and controls provided written informed consent prior to enrollment in the study. All data are available on request from the authors. Baseline demographics, co-morbidities, and NAb levels were compared between the two groups, using a chi-square test for categorical variables and an unpaired *t*-test or Wilcoxon signed-rank test (as appropriate) for continuous variables. To adjust for potential confounding effects of differences in covariates, we used case–control matching to match the two groups for age, gender, and type of vaccine with the calipmatch command in Stata. All data extraction and analyses were conducted using Stata 16.0 (StataCorp 2019, Stata Statistical Software: Release 16. College Station, TX, USA: StataCorp LLC). A two-sided *p*-value of < 0.05 was used for statistical significance.

## 3. Results

The study population included 36 patients (all females; median age: 64 years, IQR: 51–72 years) and 160 controls (all females; median age: 63 years, IQR: 60–78 years, *p* = 0.15 for age compared with patients), vaccinated during the same period. In our cohort, 30 out of 36 patients (83.3%) and 130/160 controls (81.2%) were vaccinated with mRNA vaccines (BNT162b2 and mRNA-1273), while 6/36 patients (16.7%) and 30/160 controls (18.8%) had received the AZD1222 vaccine (*p* = 0.13). Each patient and control received two doses of the assigned vaccine. The median BMI, a major risk factor for severe COVID-19 illness, was 25.8 m^2^/kg (IQR: 23–30) in the patients and 26 m^2^/kg (IQR: 23–29), in the controls (*p* = 0.44). The characteristics of the patients included in the study are depicted in Table 1. All of them had ovarian cancer treated with PARPi for a median of 4 months (range 1–17). Most patients (20 patients, 55.5%) received the PARPi olaparib, while 15 (42%) received niraparib, and one patient received rucaparib. The most frequent comorbidities in the patient group included cardiovascular disease in 44% of them, metabolic disorders in 31%, and pulmonary disease in 8%. 

**Figure 1:**
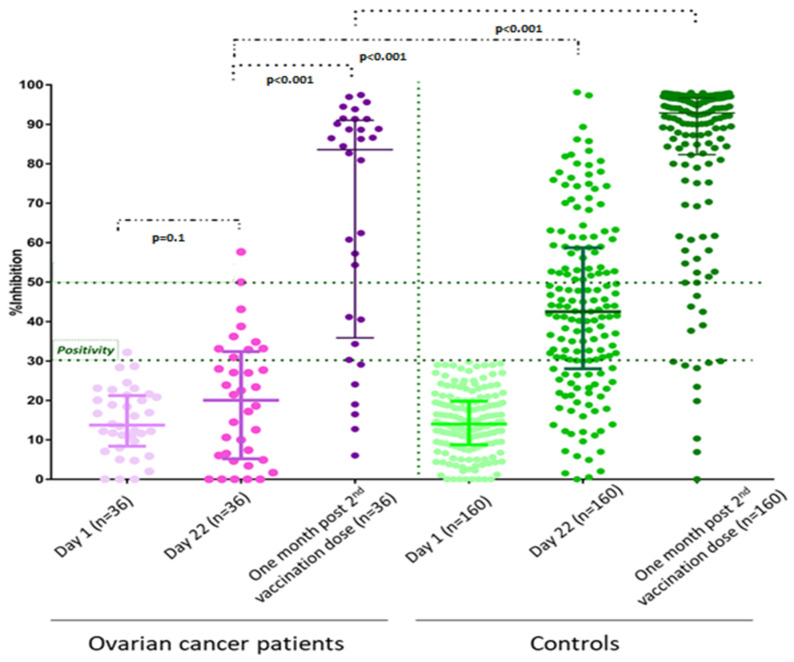
Kinetics of neutralizing antibodies in patients receiving PARPi and matched controls after vaccination with BNT162b2, AZD1222, or mRNA-1273 vaccines.

On day 1, there was no difference regarding the NAb titers between patients and controls (*p* = 0.44). None of them had a prior history of known COVID-19. After the first vaccine dose, on day 22, patients had lower NAb titers compared to controls: the median NAb inhibition titer was 20.0% (IQR: 5.5–31.9%) for patients versus 42.5% (IQR: 28.1–58.7%) for controls (*p* < 0.001) (Figure 1). More, specifically, only 10 patients (27.8%) versus 119 controls (74.4%) developed a NAb titer ≥ 30% on day 22 (*p* < 0.001). Additionally, the number of patients and controls who developed NAbs titers ≥ 50% was two (5.6%) and 57 (35.6%), respectively (*p* < 0.001). 

One month after the second vaccination dose, patients had persistently lower NAb titers compared to controls: the median NAb inhibition titer was 83.6% (IQR: 37.4–90.7%) for patients versus 92.9% (IQR: 82.4–96.6%) for controls (*p* < 0.001) (Figure 1). At that time point, 30 patients (83.3%) and 150 controls (93.8%) had developed NAb titers ≥ 30% (*p* = 0.039). Additionally, the percentage of patients and controls who developed NAb titers ≥ 50% was 72.2% and 89.4%, respectively (*p* = 0.007). No significant interaction was noted between the duration of treatment with PARPi and NAb inhibition titers among patients (*p* = 0.400). 

Regarding safety, no unexpected or severe adverse events were observed amongst the 36 patients with cancer treated with PARPi. The most frequent adverse events due to vaccination were pain at the site of injection in 22.2% of them, fever in 14%, and fatigue in 12%. There was no need to modify the oncology treatment schedule for any patient. Moreover, we did not notice a post-vaccination increase in the incidence of adverse events related to PARPi.

## 4. Discussion

The COVID-19 pandemic has posed major challenges for cancer patients, relatives and caregivers [12]. Observational studies suggest that balancing between the increased risk of COVID-19 infection when receiving treatment or the increased risk of disease progression when postponing treatment has exacerbated depression and anxiety among patients with cancer [13]. To our knowledge, we present here, for the first time, results of COVID-19 vaccination efficacy in patients receiving PARPi. Patients on treatment with PARPi that received either the BNT162b2, AZD1222, or mRNA-1273 vaccines developed lower titers of NAbs against SARS-CoV-2 up to one month after the second vaccination dose, compared to healthy controls. In cancer patients, factors such as age, the underlying disease, and the administration of immunosuppressive agents, including chemotherapy and corticosteroids, could attenuate immune response [14]. Ovarian cancer patients receive PARPi as a maintenance treatment after their disease has been put into remission with chemotherapy. In addition, these patients rarely receive immunosuppressive agents. Under this perspective, the attenuated immune response against COVID-19 vaccination recorded in our patients under treatment with PARPi, at least up to one month after vaccination completion, is of interest. It should be noted that in experimental models PARPi exert beneficial effects on SARS-CoV-2 infection [15] through various mechanisms, including the attenuation of SARS-CoV-2-induced inflammatory responses and cytokine storms as well as reducing lung fibrosis, which is a common sequel of SARS-CoV-2 infection. Therefore, repurposing these agents to treat COVID-19 has been recently suggested [16]. Moreover, PARP inhibition is recognized to affect immune cells. There is evidence of PARP involvement in T cell maturation and differentiation. PARP inhibition may additionally play a role in maintaining B cell homeostasis, differentiation, and antibody generation, while it also negatively influences the maturation and antigen-presenting function of dendritic cells [17]. Therefore, whether PARPi could also attenuate immune responses to vaccination, as suggested by our findings, deserves further evaluation.

Our results come in accordance with studies of Monin et al. and Terpos et al., reporting poor one-dose vaccine efficacy in cancer patients [18,19]. Recently, Waissengrin et al. reported the safety results of the BNT162b2 vaccine in patients with cancer treated with immune checkpoint inhibitors (ICIs) [20]. We confirm these data in our study population; amongst the 36 patients of our department who received vaccination while on treatment with PARPi, no unexpected adverse events were noted. Given the current exceptional circumstances of the COVID pandemic, cancer patients should not fall behind in the vaccination campaigns worldwide while awaiting larger, prospective trials. In any case, these results suggest that maintaining precautions against COVID-19 is essential for cancer patients, even after vaccination is completed. Emergence of new virus variants that increase transmissibility and dampen the neutralizing potency of antibodies is a significant challenge for currently existing vaccination programs. These variants could have a negative effect on vaccination efficacy [21]. In this case, our data also indicate that the administration of a third dose after a certain time would possibly be beneficial in order to generate optimal immunity for this group of patients. This is in accordance with current guidance in Greece as well as other countries throughout the world that have proposed a third vaccination dose for vulnerable patients including those with cancer. Under this perspective, it is important to continually reassess the vaccines’ effectiveness against new variants of SARS-CoV-2. Meanwhile, a further follow-up of our study population is underway, which will provide integrated data for the efficacy of vaccination in cancer patients. 

In conclusion, our data indicate that SARS-CoV-2 neutralizing antibodies are statistically lower in ovarian cancer patients under treatment with PARPi in comparison to healthy controls after completion of the vaccination program. These data should be taken into consideration to prioritize these patients by local regulatory authorities for a third vaccination dose. 

## Figures and Tables

**Table 1:** Patient characteristics.

#	SEX	AGE	BMI	TYPE OF CANCER	TYPE OF THERAPY	MONTHS ON TREATMENT	COMORBIDITIES	VACCINE	ADVERSE EVENTS
1	F	82	28.1	Ovarian cancer	Niraparib	2	Hypertension, Dyslipidemia	BNT162b2	None
2	F	77	34.3	Ovarian cancer	Olaparib	13	Breast Cancer, Dyslipidemia, Osteopenia, Diabetes	BNT162b2	None
3	F	60	30.4	Ovarian cancer	Olaparib	6	None	AZD12222	Pain at the site of injection, Fatigue
4	F	75	34.5	Ovarian cancer	Olaparib	16	Hypertension, Dyslipidemia, Chronic Obstructive Pulmonary Disease	mRNA-1273	Fever
5	F	60	26.2	Ovarian cancer	Olaparib	13	Pulmonary Embolism	AZD12222	None
6	F	47	34.2	Ovarian cancer	Niraparib	2	None	BNT162b2	None
7	F	51	22.8	Ovarian cancer	Olaparib	14	Hyperthyroidism	BNT162b2	Pain at the site of injection
8	F	42	24.5	Ovarian cancer	Olaparib	1	None	BNT162b2	Pain at the site of injection
9	F	75	28.1	Ovarian cancer	Olaparib	2	Dyslipidemia, Osteoporosis	mRNA-1273	None
10	F	57	23.4	Ovarian cancer	Olaparib	11	Hypertension, Dyslipidemia, Hypothyroidism	BNT162b2	Fever, Pain at the site of injection
11	F	68	26	Ovarian cancer	Olaparib	13	Arthritis	BNT162b2	None
12	F	56	23.6	Ovarian cancer	Niraparib	4	Hypothyroidism	BNT162b2	Pain at the site of Injection, Numbness of upper extremity
13	F	64	23.7	Ovarian cancer	Niraparib	1	Hypertension	AZD12222	None
14	F	68	21.8	Ovarian cancer	Niraparib	4	Dyslipidemia, Hypothyroidism, Depression	BNT162b2	None
15	F	57	44.9	Ovarian cancer	Niraparib	8	Hypothyroidism	BNT162b2	None
16	F	74	25.6	Ovarian cancer	Niraparib	2	Rheumatoid Arthritis, Asthma, Atrial Fibrillation	BNT162b2	Fever, Leg pain
17	F	70	32.7	Ovarian cancer	Olaparib	1	Glaucoma, Osteoporosis, Hypertension	BNT162b2	None
18	F	74	20.8	Ovarian cancer	Olaparib	17	Hypothyroidism	BNT162b2	None
19	F	71	21.6	Ovarian cancer	Niraparib	2	Dyspipidemia, Glaucoma	BNT162b2	None
20	F	79	21.4	Ovarian cancer	Niraparib	10	Dyspipidemia, Osteoporosis, Irritable Bowel, Gastritis, Deep Vein Thrombosis	BNT162b2	None
21	F	74	26	Ovarian cancer	Niraparib	4	Gastritis, Aneurysm, Hypertension	BNT162b2	None
22	F	67	23.4	Ovarian cancer	Olaparib	5	None	BNT162b2	None
23	F	73	29.3	Ovarian cancer	Olaparib	16	None	BNT162b2	Pain at the site of injection
24	F	44	27.7	Ovarian cancer	Olaparib	1	None	BNT162b2	Fatigue
25	F	70	22.5	Ovarian cancer	Olaparib	3	Hypothyroidism	BNT162b2	Fatigue
26	F	53	32	Ovarian cancer	Olaparib	1	Dyslipidemia, Hypothyroidism, Depression	BNT162b2	Pain at the site of injection
27	F	51	24	Ovarian cancer	Olaparib	4	Hypothyroidism	BNT162b2	None
28	F	48	26.2	Ovarian cancer	Olaparib	10	None	BNT162b2	None
29	F	62	25	Ovarian cancer	Niraparib	1	Dyslipidemia, Hypothyroidism	mRNA-1273	None
30	F	46	28.1	Ovarian cancer	Rucaparib	15	None	BNT162b2	Headache
31	F	47	19.5	Ovarian cancer	Niraparib	7	None	BNT162b2	None
32	F	64	21.8	Ovarian cancer	Niraparib	3	None	BNT162b2	None
33	F	52	17.5	Ovarian cancer	Niraparib	1	Hypothyroidism	BNT162b2	Fever
34	F	70	27.4	Ovarian cancer	Olaparib	3	Dyslipidemia	BNT162b2	Pain at the site of injection
35	F	67	27.9	Ovarian cancer	Olaparib	17	Hypothyroidism	BNT162b2	Fever
36	F	50	21	Ovarian cancer	Niraparib	13	None	BNT162b2	None

## Data Availability

The data presented in this study are available on request from the corresponding author.

## References

[1] Sung H., Ferlay J., Siegel R.L., Laversanne M., Soerjomataram I., Jemal A., Bray F. (2021). Global Cancer Statistics 2020: GLOBOCAN Estimates of Incidence and Mortality Worldwide for 36 Cancers in 185 Countries. CA. Cancer J. Clin..

[2] Yuki K., Fujiogi M., Koutsogiannaki S. (2020). COVID-19 pathophysiology: A review. Clin. Immunol..

[3] Rodriguez G.M., Ferguson J.M., Kurian A., Bondy M., Patel M.I. (2021). The Impact of COVID-19 on Patients with Cancer: A National Study of Patient Experiences. Am. J. Clin. Oncol..

[4] Yang L., Chai P., Yu J., Fan X. (2021). Effects of cancer on patients with COVID-19: A systematic review and meta-analysis of 63,019 participants. Cancer Biol. Med..

[5] Polack F.P., Thomas S.J., Kitchin N., Absalon J., Gurtman A., Lockhart S., Perez J.L., Pérez Marc G., Moreira E.D., Zerbini C. (2020). Safety and Efficacy of the BNT162b2 mRNA Covid-19 Vaccine. N. Engl. J. Med..

[6] Voysey M., Clemens S.A.C., Madhi S.A., Weckx L.Y., Folegatti P.M., Aley P.K., Angus B., Baillie V.L., Barnabas S.L., Bhorat Q.E. (2021). Safety and efficacy of the ChAdOx1 nCoV-19 vaccine (AZD1222) against SARS-CoV-2: An interim analysis of four randomised controlled trials in Brazil, South Africa, and the UK. Lancet.

[7] Yi M., Dong B., Qin S., Chu Q., Wu K., Luo S. (2019). Advances and perspectives of PARP inhibitors. Exp. Hematol. Oncol..

[8] Mateo J., Lord C.J., Serra V., Tutt A., Balmaña J., Castroviejo-Bermejo M., Cruz C., Oaknin A., Kaye S.B., de Bono J.S. (2019). A decade of clinical development of PARP inhibitors in perspective. Ann. Oncol. Off. J. Eur. Soc. Med. Oncol..

[9] Yélamos J., Moreno-Lama L., Jimeno J., Ali S.O. (2020). Immunomodulatory Roles of PARP-1 and PARP-2: Impact on PARP-Centered Cancer Therapies. Cancers.

[10] Terpos E., Trougakos I.P., Apostolakou F., Charitaki I., Sklirou A.D., Mavrianou N., Papanagnou E.-D., Liacos C.-I., Gumeni S., Rentziou G. (2021). Age-dependent and gender-dependent antibody responses against SARS-CoV-2 in health workers and octogenarians after vaccination with the BNT162b2 mRNA vaccine. Am. J. Hematol..

[11] Tan C.W., Chia W.N., Qin X., Liu P., Chen M.I.-C., Tiu C., Hu Z., Chen V.C.-W., Young B.E., Sia W.R. (2020). A SARS-CoV-2 surrogate virus neutralization test based on antibody-mediated blockage of ACE2-spike protein-protein interaction. Nat. Biotechnol..

[12] Mitra M., Basu M. (2020). A Study on Challenges to Health Care Delivery Faced by Cancer Patients in India During the COVID-19 Pandemic. J. Prim. Care Community Health.

[13] Ayubi E., Bashirian S., Khazaei S. (2021). Depression and Anxiety Among Patients with Cancer During COVID-19 Pandemic: A Systematic Review and Meta-analysis. J. Gastrointest. Cancer.

[14] Hiam-Galvez K.J., Allen B.M., Spitzer M.H. (2021). Systemic immunity in cancer. Nat. Rev. Cancer.

[15] Curtin N., Bányai K., Thaventhiran J., Le Quesne J., Helyes Z., Bai P. (2020). Repositioning PARP inhibitors for SARS-CoV-2 infection (COVID-19); a new multi-pronged therapy for acute respiratory distress syndrome?. Br. J. Pharmacol..

[16] Capoluongo E. (2020). PARP-inhibitors in a non-oncological indication as COVID-19: Are we aware about its potential role as anti-thrombotic drugs? The discussion is open. Biomed. Pharmacother..

[17] Lee E.K., Konstantinopoulos P.A. (2020). PARP inhibition and immune modulation: Scientific rationale and perspectives for the treatment of gynecologic cancers. Ther. Adv. Med. Oncol..

[18] Monin L., Laing A.G., Muñoz-Ruiz M., McKenzie D.R., Del Molino Del Barrio I., Alaguthurai T., Domingo-Vila C., Hayday T.S., Graham C., Seow J. (2021). Safety and immunogenicity of one versus two doses of the COVID-19 vaccine BNT162b2 for patients with cancer: Interim analysis of a prospective observational study. Lancet. Oncol..

[19] Terpos E., Zagouri F., Liontos M., Sklirou A.D., Koutsoukos K., Markellos C., Briasoulis A., Papanagnou E.-D., Trougakos I.P., Dimopoulos M.-A. (2020). Low titers of SARS-CoV-2 neutralizing antibodies after first vaccination dose in cancer patients receiving checkpoint inhibitors. J. Hematol. Oncol..

[20] Waissengrin B., Agbarya A., Safadi E., Padova H., Wolf I. (2021). Short-term safety of the BNT162b2 mRNA COVID-19 vaccine in patients with cancer treated with immune checkpoint inhibitors. Lancet. Oncol..

[21] Krause P.R., Fleming T.R., Longini I.M., Peto R., Briand S., Heymann D.L., Beral V., Snape M.D., Rees H., Ropero A.-M. (2021). SARS-CoV-2 Variants and Vaccines. N. Engl. J. Med..

